# E3 ligase mahogunin (MGRN1) influences amyloid precursor protein maturation and secretion

**DOI:** 10.18632/oncotarget.20143

**Published:** 2017-08-10

**Authors:** Stefano Benvegnù, Tina Wahle, Carlos G. Dotti

**Affiliations:** ^1^ Department of Molecular Neurobiology, Centro de Biología Molecular ”Severo Ochoa”, CSIC-UAM, Madrid, Spain; ^2^ IUF - Leibniz Research Institute for Environmental Medicine, Düsseldorf, Germany

**Keywords:** Alzheimer's disease, APP, amyloid beta, mahogunin, trafficking, Gerotarget

## Abstract

Altered processing of the Amyloid Precursor Protein (APP) is a well-recognized central pathogenic mechanism in Alzheimer's Disease (AD), and regulation of APP processing is a major focus of research in the AD field. However, how age-associated cellular and molecular changes contribute to changes in the amyloidogenic processing of APP have not been extensively clarified so far. We here provide evidence that the processing of APP is influenced by the e3 ubiquitin ligase Mahogunin (MGRN1), a neuroprotective molecule whose levels decrease with aging. Specifically, the expression of MGRN1 inhibits the maturation of APP by sequestering it in the secretory pathway. This sequestration significantly delayed the proteolytic processing of APP, resulting in a reduced β-amyloid (Aβ) peptide release into the extracellular environment. Accordingly, a reduction of MGRN1 levels in hippocampal neurons, as it occurs during physiological aging, leads to an increased Aβ40 and Aβ42 release. We therefore propose that age contributes to the amyloidogenic processing of APP by altering its intracellular trafficking along the secretory pathway due in part to the down-regulation of MGRN1.

## INTRODUCTION

The pathogenesis of Alzheimer's disease (AD) is linked to the proteolytic processing of the amyloid precursor protein (APP) to the amyloidogenic peptide Aβ. Aβ is a 39-43-amino acid peptide that is the main constituent of the parenchymal and cerebrovascular deposits characteristic of AD [1]. Aβ itself is generated by sequential cleavage of APP by β- and γ-secretase. APP, a type I transmembrane protein, is synthesized in the endoplasmic reticulum (ER) and transported through the Golgi/trans-Golgi network (TGN) apparatus where it is subjected to post-translational modifications such as glycosylation, sulfation, and phosphorylation [2]. APP exists as immature (N-glycosylated) and mature (N- and O-glycosylated) species. While the immature APP localizes in the ER and cis-Golgi, the mature APP localizes in compartments following TGN. APP can be transported to the cell surface in TGN-derived secretory vesicles. At the plasma membrane APP is either cleaved by α-secretase in a non-amyloidogenic fashion or re-internalized within clathrin-coated vesicles to endo-/lysosomal compartments [3]. Once APP reaches the endosomes it can be processed by β-secretase, be recycled back to cell surface or be delivered to the lysosome for degradation [3, 4]. The amyloidogenic processing of APP occurs by sequential cleavage by β- and γ-secretase to release Aβ peptide. However, the complete molecular mechanism(s) and cellular compartment(s) involved in APP cleavage and Aβ production are still under debate [5]. Proteolytic processing of APP was shown to occur in different sites all through the secretory and endocytic pathways. In any case, amyloidogenic processing mostly occurs following transition through the Golgi apparatus and in endosomal compartments, where acidic conditions favors optimal activity of β-secretase [6]. APP trafficking is tightly regulated as it travels through secretory and endo/lysosomal compartments of the cell and alteration in APP trafficking is often accompanied by changes in APP processing. Factors that regulate APP trafficking and processing can, in turn, regulate also Aβ production and release in the extracellular milieu. The identification of such factors is therefore important to understand the pathogenesis of AD and its consequences.

Among the different risk factors for AD, aging is the best known [7]. The risk of developing AD doubles every 5 years after age 65 [8]. Mechanisms that, directly or indirectly, participate in neuronal weakening during physiological aging and thus confer increasing neuronal susceptibility to cytotoxic stress, can in turn render neurons more prone to the subsequent development of an age-related disease, eventually switching physiological aging to a pahtological aging phenotype. We recently described that aging triggers the depletion of the E3 ubiquitin ligase *Mahogunin* (MGRN1) and the underlying molecular mechanisms [9]. Mahogunin/MGRN1 plays important functions in cellular and neuronal protection: *i.e*. it protects neurons against oxidative and endoplasmic reticulum stress, against cytosolic prions and polyglutamine neurotoxic aggregates, indicating that it may play a protective role in different neurodegenerative conditions [10–12]. However, so far, no data was reported about a potential role of MGRN1 in AD. This moved us to investigate a potential MGRN1 function in the context of AD.

## RESULTS

**Figure 1:**
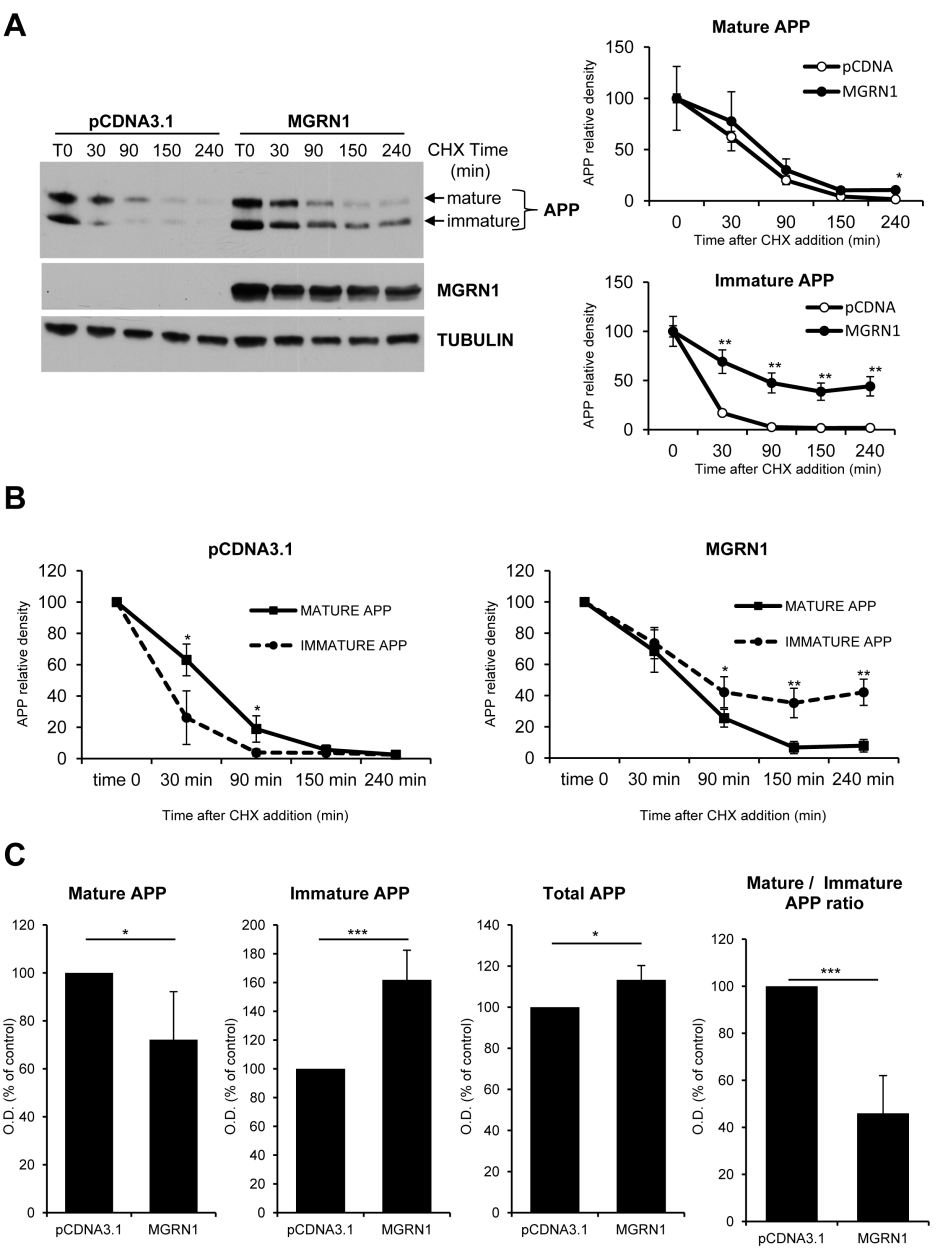
MGRN1 inhibits APP maturation in transfected HEK293 cells **A.** Degradation rate of mature and immature levels of APP in absence or presence of MGRN1. Cells were transfected either with empty pCDNA3.1- or with MGRN1 plasmid, and then treated with CHX and collected at the indicated time points (in minutes). On the right, quantification of mature APP (upper graph) and immature APP (lower graph) from 4 independent experiments indicating how MGRN1 affects significantly the degradation rate of immature APP. Tubulin was used as loading control. Data are mean ± s.d. **B.** Quantification from 4 independent experiments of the degradation rate of mature APP *versus* immature APP in absence (left graph) of presence (right graph) of MGRN1. Data are mean ± s.d. **C.** Quantification of the steady-state levels of APP in absence (pCDNA3.1) or presence of MGRN1. MGRN1 decreased the levels of mature APP (first panel), increased the levels of immature APP (second panel) and total APP (third panel), and decreased the ratio of mature APP to immature APP (fourth panel) compared with control. Quantification from 5 independent experiments. Data are mean ± s.d.

In addition to its neuroprotective roles (see Introduction), the E3ubiquitin ligase MGRN1/Mahogunin regulates endomembrane fusion processes [13]. We have recently demonstrated that MGRN1/Mahogunin decreases in old hippocampal neurons [9] and numerous works have stressed the importance of the neurons´ endomembrane system in the amyloidogenic processing of APP [6, 14]. Hence, we decided to investigate whether or not age-associated increase in amyloid processing was functionally related to the age-associated Mahogunin downregulation. For this purpose, we first studied the effect of over-expressing MGRN1 in cycloheximide-treated HEK293 cells stably expressing human wild-type APP695. Trafficking through the Golgi was monitored by the transition of APP from an immature form, which migrates faster on SDS/PAGE, to a mature more slowly migrating form that has undergone O-linked glycosylation. In cells overexpressing MGRN1, the mature form of APP is degraded at approximately similar rates; however, the immature form of APP persists throughout the time course longer than in mock-transfected cells (Figure 1A). Moreover, in control conditions the mature form of APP is degraded at a slower rate than its corresponding immature form (Figure 1B, left panel). On the contrary, the presence of MGRN1 shifts the degradation of APP, inducing a higher retention of the immature APP form and a faster degradation of the mature form (Figure 1B, right panel). At steady state, MGRN1 decreased the levels of mature APP and concomitantly increased the levels of immature APP and total APP in comparison to control. In addition, MGRN1 decreased the ratio of mature APP to immature APP compared with control, suggesting that MGRN1 is retarding APP maturation (Figure 1C). As a whole, all these results suggest that MGRN1 may modulate the degree of APP maturation by sequestering immature APP in secretory compartments before its complete passage through the final Golgi compartments.

**Figure 2:**
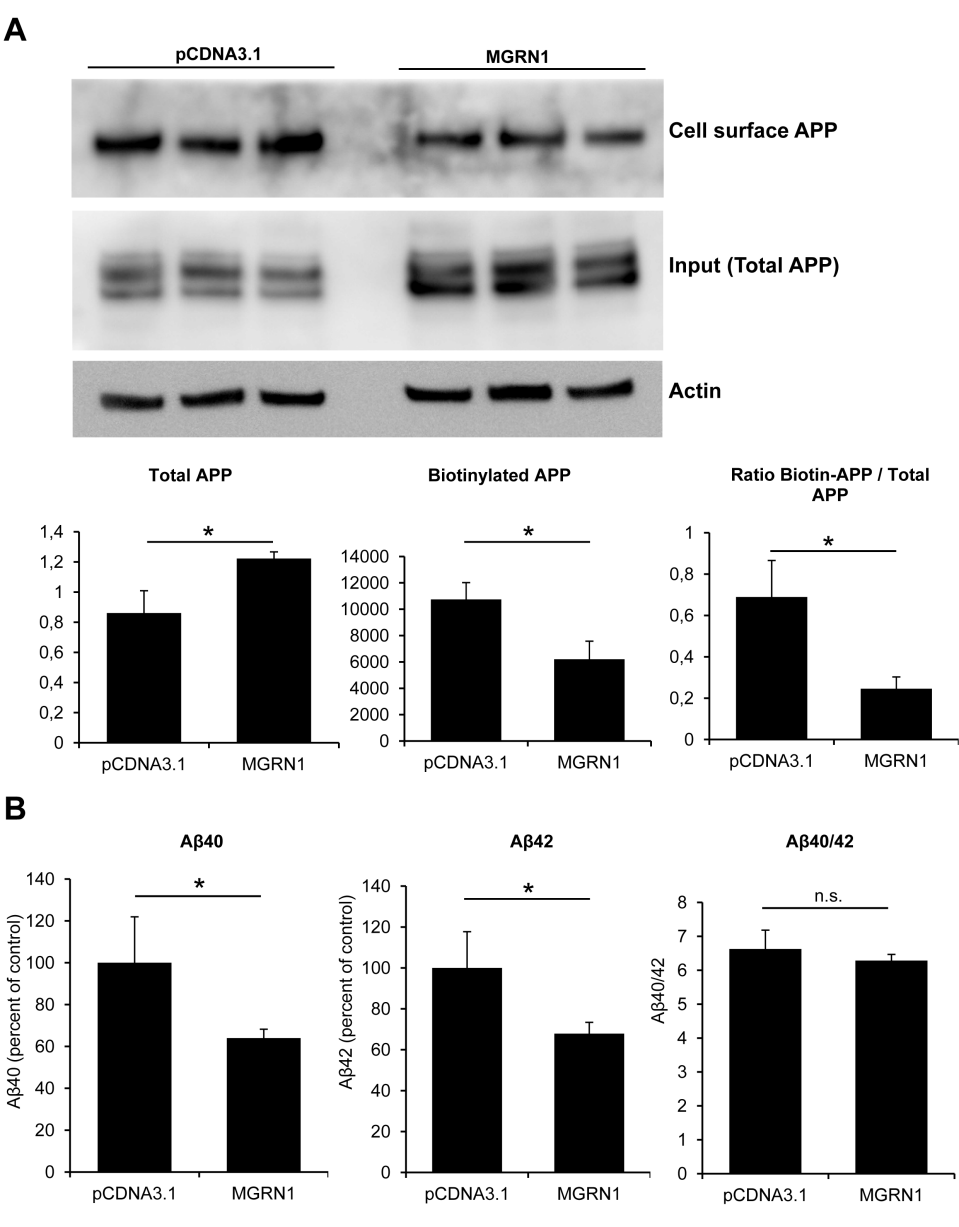
MGRN1 decreases cell-surface APP and Aβ secretion **A.** Biotinylation experiments to examine cell-surface expression of APP in cells expressing MGRN1 or transfected with empty pCDNA3.1- vector. Triplicate experiments show how cells expressing MGRN1 show a decreased level of APP localized at the cell surface. On the bottom, quantification of total APP (left graph), biotinylated APP (central graph) and the ratio between biotinylated APP and total APP (right part). Quantification from 3 independent experiments. Data are mean ± s.d. **B.** ELISA experiments performed on the conditioned media of mock- and MGRN1-transfected HEK293 cells to detect Aβ. MGRN1 decreased the levels of both Aβ40 (left graph) and of Aβ42 (central graph), while the Aβ40/42 ratio is not affected (right graph). Quantification from 3 independent experiments. Data are mean ± s.d.

Sequestration of APP in the secretory pathway would prevent trafficking of APP to the cell surface. To explore this possibility, the plasma membrane levels of APP were quantified by cell-surface biotinylation experiments. In agreement with reduced maturation, MGRN1 overexpression caused a significant decrease in plasma membrane-associated APP (Figure 2A). In order to further evaluate that APP processing is retarded in the MGRN1 condition, we quantified by sandwich ELISA the amount of Aβ40 and Aβ42, the major Aβ isoforms, in the medium of MGRN1-overexpressing HEK293 cells stably expressing human wild-type APP(695). Production of both Aβ40 and Aβ42 was found to be significantly decreased by the expression of MGRN1 (Figure 2B). We identified no changes, mediated by the expression of MGRN1, in the expression levels of the gamma-secretase complex members Nicastrin, Presenilin-1 and Aph-1alpha (Supplementary Figure 1A), indicating that a potential involvement of MGRN1 in the gamma-secretase-mediated metabolism of APP is to be considered unlikely. We additionally identified that MGRN1 expression induces an increase in the expression levels of the neuroprotective product secreted APP-alpha in the conditioned medium (Supplementary Figure 1B), a result in agreement with our observation that MGRN1 expression induces an attenuation of the pro-amyloidogenic processing of APP and a consequent reduced production of Aβ.

**Figure 3:**
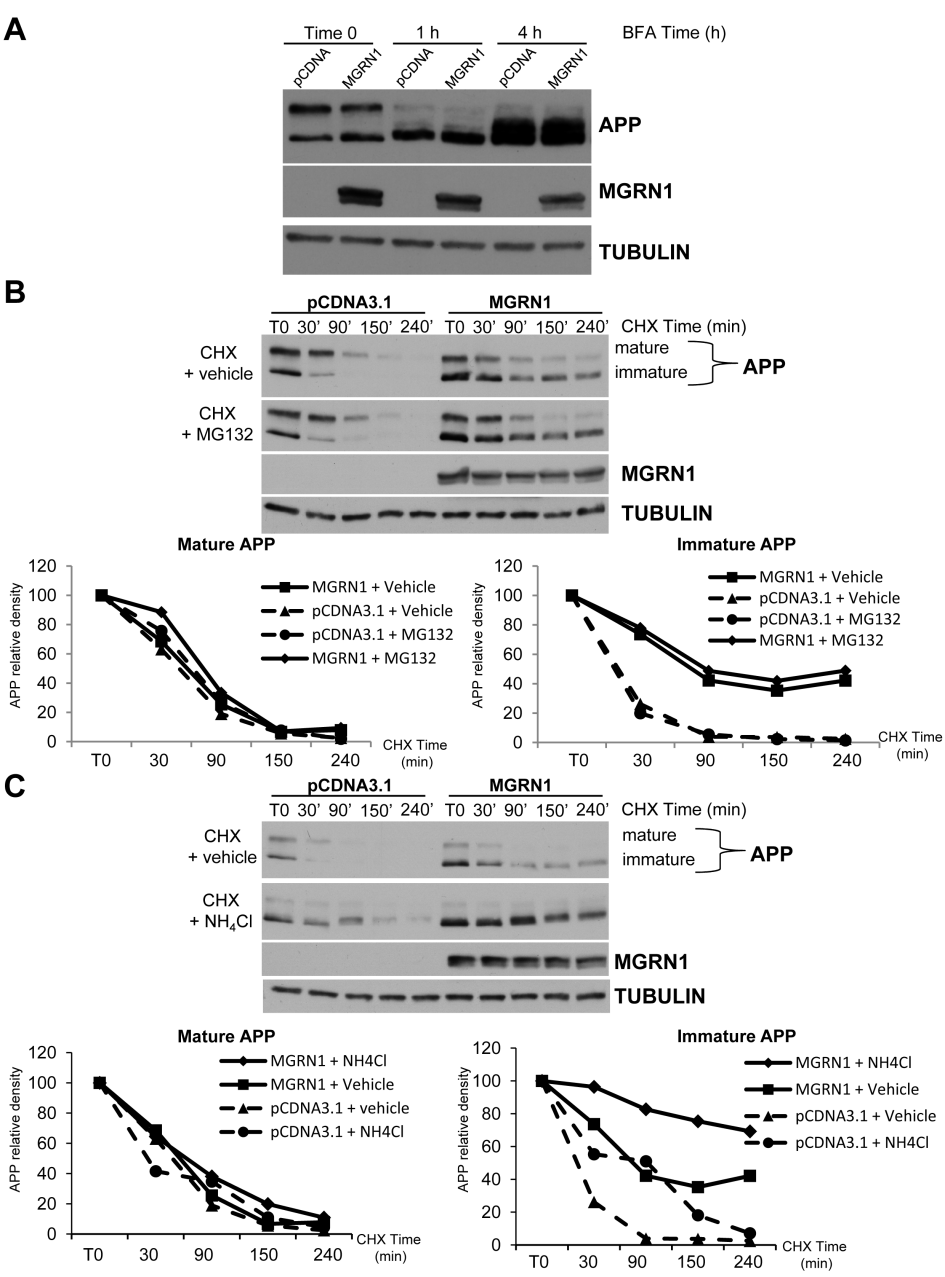
MGRN1 inhibits nonlysosomal degradation of APP **A.** HEK293 cells stably overexpressing human wild type APP695 were either mock- or MGRN1-transfected, and then treated with Brefeldin A (BFA) for 1 hour or 4 hours. MGRN1 presence is not affecting the formation of intermediate APP isoforms. Tubulin was used as loading control. Representative of *n* = 3 experiments. **B.** Time course of APP degradation after addition of cycloheximide in the absence (“+ vehicle”, first lane) or presence (second lane) of the proteasomal inhibitor MG132. Tubulin was used as loading control. Representative of *n* = 3 experiments. **C.** Time course of APP degradation after cycloheximide addition in the absence (“+ vehicle”, first lane) or presence (second lane) of the lysosomal inhibitor NH_4_Cl. Tubulin was used as loading control. Representative of *n* = 3 experiments.

One possible mechanism by which MGRN1 may increase immature APP levels is that it may also affect APP metabolism at the level of the endoplasmic reticulum. In order to study this possibility, we took advantage of treatment with Brefeldin A (BFA). BFA is an agent that disassembles the Golgi complex and redistributes proteins into the ER. BFA treatment has been shown to induce the formation of intermediate APP isoforms [15]. Therefore, if MGRN1 would affect the levels of intermediate APP isoforms induced by BFA treatment, this would suggest that MGRN1 could act before the Golgi, *i.e*. at the endoplasmic reticulum. However, BFA-induced formation of intermediate APP isoforms was not significantly modified either in the presence or absence of MGRN1, at earlier time (1 hour treatment) and at longer time (4 hours treatment) (Figure 3A). These results indicate that MGRN1 does not afftect APP metabolism in the ER, and corroborate a role for MGRN1-regulated APP processing in the secretory pathway at the level of the Golgi.

To further investigate whether MGRN1 regulates the proteolysis of APP, its ability to modulate the half-life of APP was examined in the presence of the proteasome inhibitor MG132 or the lysosomal inhibitor NH_4_Cl. As shown in Figure 3B (left part), APP was rapidly degraded in the absence of MG132, and this degradation was slightly attenuated by proteasome inhibition. MGRN1 overexpression (right part) had little effect on APP degradation in the presence of MG132. When the cells were treated with the lysosomal inhibitor ammonium chloride (NH_4_Cl), the degradation rate of immature APP was significantly delayed (Figure 3C, left part). These results are consistent with previous findings indicating that a large part of APP is degraded in acidic compartments, including lysosomes [16, 17]. Overexpression of MGRN1 strongly inhibited immature APP degradation in the presence of the lysosome inhibitor (Figure 3C, right part). This result suggests that nonlysosomal degradation of APP (as determined by half-life in the presence of NH_4_Cl) is significantly inhibited by MGRN1 overexpression.

**Figure 4:**
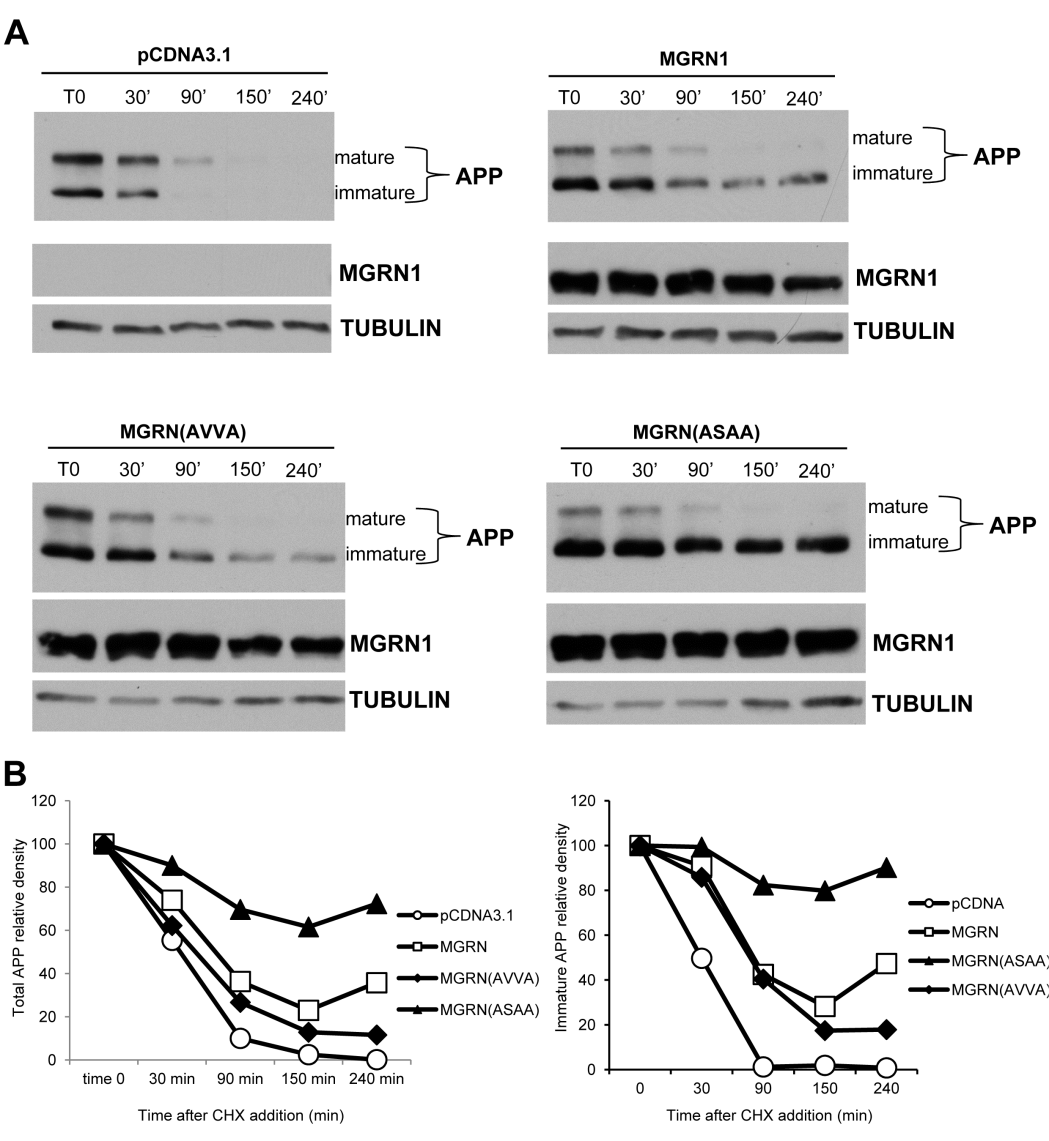
MGRN1 mutational studies **A.** HEK293 cells stably overexpressing human wild type APP695 were transfected either with empty plasmid (pCDNA3.1, upper left panel), or with MGRN1 plasmid (upper right panel), with the catalytically inactive MGRN(AVVA) mutant (lower left panel), or with MGRN1 mutant lacking the binding site to TSG101, MGRN(ASAA) mutant (lower right panel). Then the time course of APP degradation was monitored after addition of cycloheximide (CHX) at the indicated times. Tubulin was used as loading control. Representative of *n* = 3 experiments. **B.** Quantification of band intensities of APP in an experiment representative of three separates determinations.

We next tested whether MGRN1 E3 catalytic activity is necessary for delaying APP maturation. For this purpose, we took advantage of a catalytically-inactive form of MGRN1 (MGRN1_AVVA_). The MGRN1_AVVA_ mutant was created by replacing the cysteine residues at positions 278 and 281 of the MGRN1 RING finger domain. This mutation has previously been shown, by us and other groups, to inactivate the catalytic activity of MGRN1 [12, 18]. We transfected HEK293-APP(695) cells with either widltype MGRN1 or with MGRN1_AVVA_ mutant, and then we followed APP proteolysis treating cells with the cycloheximide. MGRN1_AVVA_ mutant expression (lower left panel) had the same effect as widltype MGRN1 expression on APP proteolysis, suggesting that effect of MGRN1 on immature APP are independent from its E3 catalytic activity. MGRN1 was also shown to bind and ubiquitinate TSG101, a key component of the ESCRT-I complex, and to play an essential role in endosome-to-lysosome trafficking [19]. It has been recently shown how ESCRT complexes regulate amyloid precursor protein sorting in multivesicular bodies, and specifically that depletion of TSG101 inhibited targeting of APP to exosomes and the subsequent delivery to lysosomes, resulting in increased intracellular Aβ accumulation and consequent decreased Aβ secretion [20]. In particular, MGRN1 interacts with and multi-ubiquitinates TSG101 to mediate vesicular fusion with lysosomes, and the perturbation of the interacting region of MGRN1 (a PSAP motif) with TSG101 compromises vesicle fusions with lysosomes, perturbing eventually the target protein(s) degradation [13]. We decided therefore to study the effect of a MGRN1 mutant which lacks the binding site to TSG101 (PSAP - ASAA). In the presence of MGRN(ASAA) mutant, APP is less degraded than in the presence of wild-type MGRN1. This result is even more evident at the level of the immature form of APP (Figure 4B, right panel), and it is similar to the effect obtained with wild-type MGRN1 in the presence of the lysosomal inhibitor NH_4_Cl.

These results confirm that a correct functioning of ESCRT complex is important for the physiological processing and degradation of APP.

**Figure 5:**
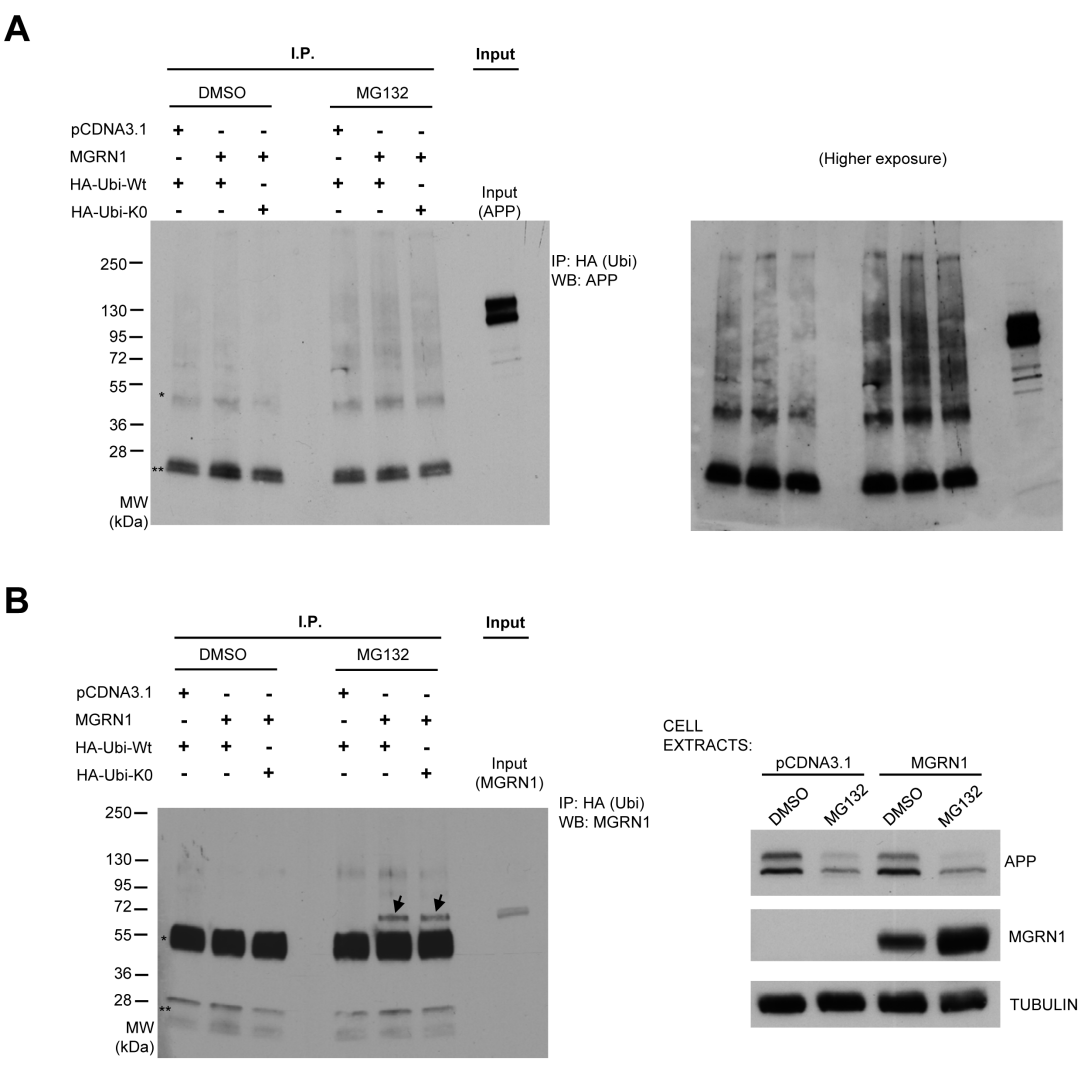
MGRN1 does not influence APP ubiquitination **A.** HEK293 cells stably overexpressing human wild type APP695 were transfected with an empty plasmid or with MGRN1, and then with either HA-tagged wild-type ubiquitin (HA-Ubi-Wt) or with a lysine-less ubiquitin mutant (HA-Ubi-K0) able to promote only monoubiquitination. Cell extracts were then immunoprecipitated (“I.P.”) with anti-HA antibody, and then revealed against APP. One well on the right (“Input”) was loaded with cell extract in order to identify to molecular weight of APP. One asterisk (*) denote non-specific IgG heavy chains band, while two asterisks (**) denote non-specific IgG light chains band. Molecular weights are shown on the left. On the right, a higher exposure panel of the same western blot is shown. **B.** The membrane was re-incubated with anti-MGRN1 antibody. Two definite bands, denoted by arrows, appear on the wells relative to extracts of cells transfected with MGRN, ubiquitin and then treated with MG132. This result indicates that MGRN1 is monoubiquitinated, and confirms previous findings. The inputs of the cell extracts are shown on the right panel. Tubulin was used as loading control. Representative of *n* = 3 experiments.

We next tested whether MGRN1 regulates APP trafficking by stimulating APP ubiquitination. While polyubiquitination signals are mostly used for target protein degradation, monoubiquitinations are implicated in many nondegradative processes, including protein trafficking [21]. Several lines of evidence suggest that ubiquitination of APP regulates its trafficking. For example, ubiquitination at APP K688 results in APP being sequestered in the early secretory pathway, mainly within the Golgi apparatus [22]. In order to study whether MGRN1 can promote APP mono- or poly-ubiquitination, HEK293T cells were transfected either with an empty plasmid or with MGRN1, in addition to either HA-tagged wild-type ubiquitin (HA-Ubi-Wt) or a lysine-less ubiquitin mutant (HA-Ubi-K0) able to promote only monoubiquitination. Immunoprecipitation with anti-HA antibody and subsequent western blot against APP revealed however that MGRN1 had no detectable effect on APP ubiquitination status, neither in control conditions nor under proteasome inhibition treatment (to allow accumulation of ubiquitinated proteins) (Figure 5A, 5B).

**Figure 6:**
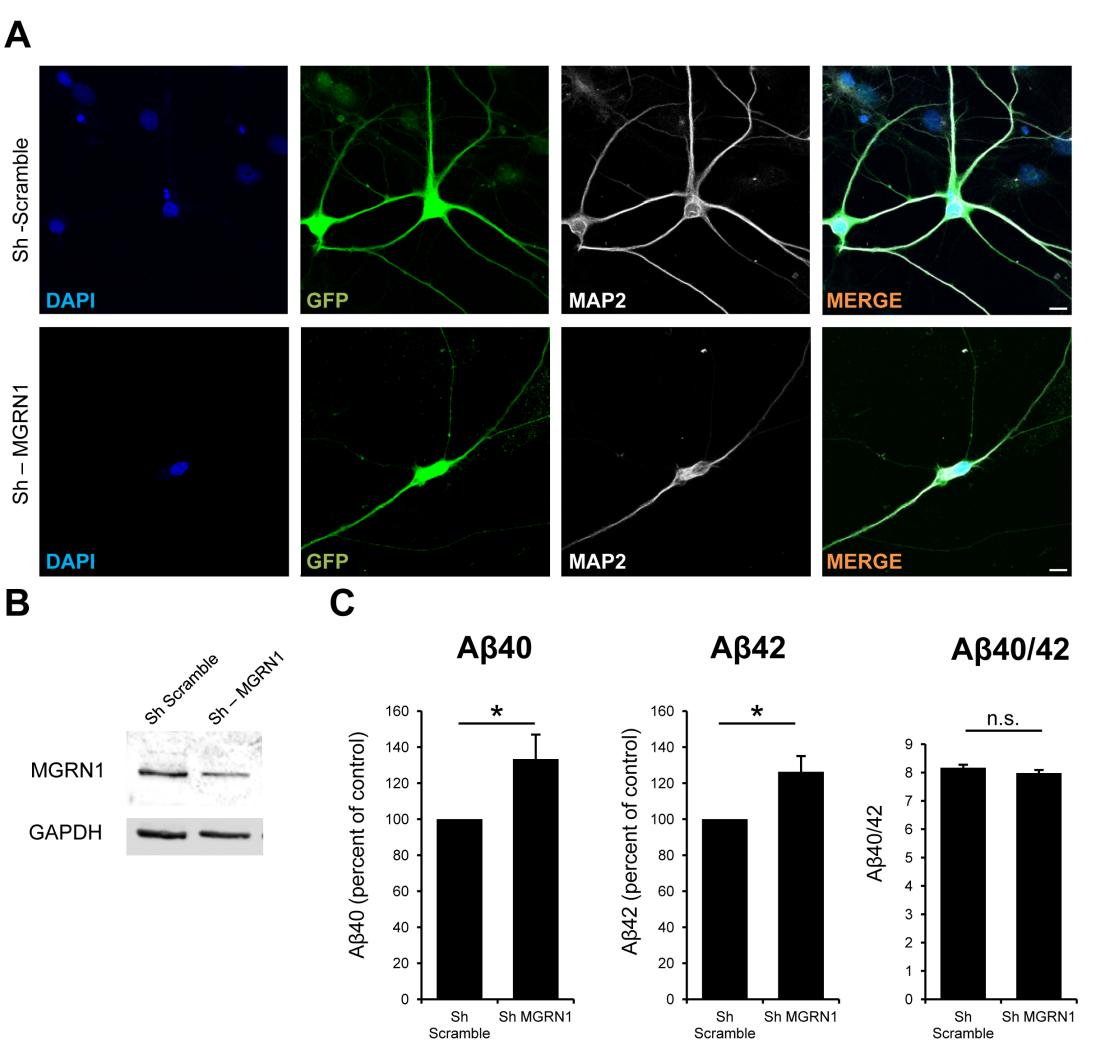
MGRN1 knockdown increases Aβ secretion in neurons **A.** Representative images of primary rat hippocampal neurons infected at 6 days in vitro (DIV) with lentiviral particles bearing either an sh-scramble control sequence (upper panles) or a Sh directed against endogenous rat *Mgrn1*. GFP channel, indicative of positive infection, and MAP2 channel, marker of mature neurons, are also shown. No gross morphological alterations are detected in Mgrn1-depleted neurons compared to Sh-scramble infected neurons. Scale bar = 10μm. **B.** Western blot analysis of the extracts from the same primary rat hippocampal neurons as in (A). The western blot analysis shows a reduction of MGRN1 intensity, indicative of knockdown of the protein. The positive knockdown of the protein with the lentiviral particles used in this study was already demonstrated elsewhere [9]. **C.** The conditioned medium of the same neurons as in (B) were analyzed with ELISA for their Aβ content. Knockdown of MGRN1 induces an increase in both Aβ40 (first panel) and Aβ42 (second panel), while the Aβ40/42 ratio is not affected (right panel) (n = 5, Data are mean ± s.e.m.).

If upregulation of MGRN1 reduces Aβ levels as shown above, theoretically a reduction of its levels as we observed in aging [9] would consequently increase Aβ levels. We then decided to knockdown MGRN1 levels in rat hippocampal neurons *in vitro,* which express endogenous levels both of MGRN1 and of APP, with lentiviral vectors expressing a sh-RNA construct against rat *Mgrn1* gene (Figure 6A, 6B). MGRN1 knockdown led to an increase of both Aβ40 and Aβ42 in the conditioned medium of the infected neurons compared to sh-scramble control infected neurons, leaving Aβ40/Aβ42 ratio unaltered (Figure 6C). These results confirm our finding on transfected HEK293 cells, and corroborate a role for MGRN1 in the final production and release of extracellular Aβ.

## DISCUSSION

We here describe that MGRN1 expression delays APP maturation, most probably by sequestering it in the Golgi apparatus. APP is therefore less transported to the cell surface or reinternalized into endosomal/lysosomal compartements where it can be processed. As a final consequence, this delayed APP maturation and processing exerted by MGRN1 leads to a decreased Aβ peptide release into the extracellular milieu. Accordingly, a reduction of MGRN1 levels in neuronal culture leads to an increase in Aβ peptide release. As a whole, these results indicate that MGRN1 is a strong regulator of APP processing and trafficking.

Molecular changes that occur with aging are strongly connected with AD. For example, it was already described that β-secretase BACE1 activity increases with age, and as a consequence the levels of Aβ increase [23]. Additionally, nitrotyrosination of gamma-secretase complex increases with aging, and this leads to an increase in Aβ42 levels [24]. We here described that another protein (*i.e.* MGRN1), whose levels change with aging, is associated to APP processing, and that the reduced MGRN1 levels observed with aging can influence APP metabolism towards a pro-amyloidogenic phenotype . MGRN1 is a e3 ligase; however, we did not identify any direct effect of MGRN1 on APP ubiquitination state (Figure 5), suggesting that the described effect on APP metabolism may not be a direct effect of MGRN1 on APP protein ubiquitination. MGRN1 was already shown to regulate endosomal trafficking and lysosomal fusion [13, 19]. In light of these properties, we can postulate that the effect exerted on APP metabolism may therefore be due to MGRN1 influence on vesicular trafficking. In this perspective, MGRN1 may likely function as a key quality-control molecule that limits the metabolism of APP towards its amyloidogenic processing and, as a consequence, Aβ-associated neuronal dysfunction. However, low levels of Aβ were also proposed to have physiological functions [25, 26]; indeed, as Aβ can be detected in the CSF of non-AD individuals and in the conditioned media from neuronal cell cultures [27, 28], this indicates that, on top of having a pathological role in Alzheimer's disease, Aβ can have a potential role in the normal physiology of the central nervous system, and that only after exceeding a concentration threshold (when its production and degradation are imbalanced) Aβ can be regarded as a neurotoxic molecule [25]. Therefore, from our results we cannot exclude that an enhanced Aβ release due to MGRN1 knockdown in aging could be also a kind of early physiological event for neuronal adaptation to the aging phenotype background. Moreover, as the accumulation of both Aβ40 and Aβ42 was similar in condition of MGRN1 knockdown in neuronal cultures *in vitro*, our results suggest that both short and larger (potentially more toxic) aggregate species could similarly accumulate in the aging brain following MGRN1 decrease *in vivo*. Additional experiments are therefore needed to further investigate in detail the *in vivo* relevance of the enhanced Aβ release during aging.

Considering that the ESCRT machinery has a role in targeting APP and its processing products to the lysosome for degradation, and promotes Aβ secretion [20], and that MGRN1 has a pro-stabilization effect on APP and limits Aβ secretion (this work), we can hypothesize that the effect of MGRN1 on delaying APP processing may be dependent also on cellular machineries and components other than the ESCRT complex, and that, in the end, the net effect of overexpressing MGRN1 overwhelms the effect that MGRN1 has only on TSG101 and ESCRT pro-degradative function on APP. MGRN(ASAA) mutant is not monoubiquitinating and therefore not activating TSG101 to induce ESCRT pro-degradative effect on APP, so in the presence of (MGRN)ASAA the effect of ESCRT is “switched off”, compared to overexpressing wildtype MGRN1. Therefore, we can propose that MGRN(ASAA) mutant is not playing a “double” functions for APP processing as wildtype MGRN1 does (activating TSG101 on one “pro-degradative APP” side, and functioning on other cellular machineries and components on another “pro-stabilization APP” side), but may function directly and only on the other cellular machineries responsible for APP processing. This would explain why MGRN(ASAA) mutant has an effect on APP stabilization stronger than wildtype MGRN1. Future work is needed to better clarify the interplay between MGRN1 and the ESCRT complex in the metabolism of APP.

## MATERIALS AND METHODS

### Primary cultures and cell lines

Rat primary hippocampal cultures were prepared from embryonic day 18 brains as described [29]. For biochemical analysis, 3×10^5^ neurons were plated in 35mm plastic dishes coated with poly-L-lysine (0.1 mg/mL) and containing Neurobasal medium (Life Technologies) with B27 supplement (Gibco). For immunofluorescence analysis, 7×10^4^ cells were plated onto glass coverslips coated with poly-L-lysine (1 mg/mL). Human embryonic kidney (HEK) 293 cells stably overexpressing human wild type APP695 were grown in Dulbecco's modified Eagle's minimum (DMEM) essential medium (Invitrogen) supplemented with 10% heat-inactivated fetal bovine serum (Perbio) and 100 μg/ml of penicillin/streptomycin (Invitrogen).

### Plasmids and cells transfection

The ORF of human *MGRN1* was retro-transcribed from human brain RNA with RT-PCR (SuperScript III, Invitrogen) and cloned in pcDNA3.1- vector (Invitrogen). The MGRN1 mutants described in the manuscript were generated with standard PCR techniques or with site-directed mutagenesis (QuikChange II Site-Directed Mutagenesis Kit, Agilent Technologies). pRK-HA-Ubiquitin-WT (#17608) and pRK-HA-Ubiquitin-K0 (#17603) plasmids were from Addgene. Cells were transfected with Lipofectamine 2000 (Life technologies) according to the manufacturer instructions.

### Antibodies

The following antibodies were used: APPCt C-terminal antibody (A8717, Sigma); APP 6E10 (Covance); MGRN1 (sc-134385, SantaCruz Biotechnology); Presenilin was detected with the SB129 antibody, Nicastrin with the 9C3 antibody [30], Aph1a with the B80.3 [31]. Tubulin (7291, Abcam).

### Lentiviral production and neuronal infection

Packaging plamids and Sh-RNA plamids against MGRN1 were purchased from Origene. Lentiviral particles were produced according to Origene instructions. Neurons were infected at 5-6 days in vitro (DIV) and medium was fully changed with neuronal conditioned medium after infection.

### Aβ measurements and secreted APPα in conditioned medium

ELISA kits for Aβ40 (#294-62501) and Aβ42 (#290-62601) detection were purchased from Wako. For Aβ detection in conditioned medium of HEK293, cells were transfected either with empty pCDNA3.1- plasmid or with pCDNA3.1-MGRN plasmid, and the medium was replaced with fresh medium 6 hours post-transfection. Cells were grown for another 48 hours and media were assayed for Aβ load. For Aβ detection in medium of primary hippocampal neurons, neurons were cultured as described. After infection either with sh-scramble control or with sh-MGRN lentiviral particles, neurons were cultures for another 7 DIV and then medium was collected and assayed for Aβ content. sAPPα levels from the conditioned media of transfected HEK293 cells were analyzed using western blotting with 6E10 (Covance) antibody.

### Cellular membrane preparations

HEK293 cells stably overexpressing human wild type APP695 were transfected either with empty pCDNA3.1- plasmid, or with MGRN1 plasmid. 72 hours after transfection, cells were washed with ice-cold PBS 1x, scraped in ice-cold PBS 1x and centrifuged at 1200 rpm, 3 min, 4°C. Cellular pellets were then incubated for 10 min on ice with hypotonic buffer (10mM Tris pH 7.6, 1mM EDTA, 1mM EGTA). Cells were then passed through a 1mL syringe 0,6mm diameter needle on ice, and cellular debris and nuclear fractions were removed by centrifugation at 4000rpm, 10 min, 4°C. Supernatants were then passed into new tubes, and centrifuged at 13200rpm, 10 min, 4°C to obtain membrane fractions. Membrane pellets were then resuspended in STEN-lysis buffer (1× STEN: 50 mm Tris, pH 7.6, 150 mm NaCl, 2 mm EDTA, 0.2% Nonidet P-40; STEN-lysis buffer, 1% Triton X-100, 1% Nonidet P-40, complete protease inhibitors in 1× STEN).

### Cell surface biotinylation

Cells were washed three times with ice-cold 1x PBS and incubated on ice with 1x PBS, pH 8, containing 0.5 mg/ml of EZ-link Sulfo-NHS-SS-biotin (Pierce) for 30 min. Cells were then washed three times, 5 min each, with ice-cold 1x PBS supplemented with 20 mM glycine and finally washed twice with 1x PBS. Cells were lysed as described before. Biotinylated proteins were immunoprecipitated using streptavidin-agarose.

### Ubiquitination assays and immunoblotting

Cells were transfected as described, and lysed 72 hours post-transfection in STEN lysis buffer (1× STEN: 50 mm Tris, pH 7.6, 150 mm NaCl, 2 mm EDTA, 0.2% Nonidet P-40; STEN-lysis buffer, 1% Triton X-100, 1% Nonidet P-40, complete protease inhibitors in 1× STEN) and clarified by a 30-min centrifugation at 13,200 × g. For immunoprecipitation of proteins the lysates were incubated overnight with anti-HA antibody and protein A/G-Sepharose beads (Santa Cruz Biotechnology). The day after, the beads were washed twice with STEN-NaCl (STEN buffer with 500 mm NaCl), and once with STEN buffer. Upon SDS-PAGE electrophoresis, immunoprecipitated proteins were transferred to PVDF membrane and detected with the corresponding antibodies.

### Statistical analysis

Data are expressed as the mean ± s.d or ± s.e.m. of the values from the independent experiments performed, as indicated in the corresponding figures legends. Student's t-test was used for statistical analysis of the data using GraphPad (GraphPad Software, Inc.). P values lower than 0.05 were considered significant in a 95% confidence interval. In the figures asterisks indicate P values as follows: *<0.05; **<0.01; ***<0.001.

## SUPPLEMENTARY MATERIALS FIGURES AND TABLES


