# Supplementary material for: E3 ligase mahogunin (MGRN1) influences amyloid precursor protein maturation and secretion
Source: Oncotarget. 2017 Aug 10;8(52):89439–50. doi: 10.18632/oncotarget.20143 (PMC5685682; doi:10.18632/oncotarget.20143)
Supplement: Supplementary file 1 [file oncotarget-08-89439-s001.pdf]

E3 ligase mahogunin (MGRN1) influences amyloid precursor protein maturation and secretion

SUPPLEMENTARY MATERIALS

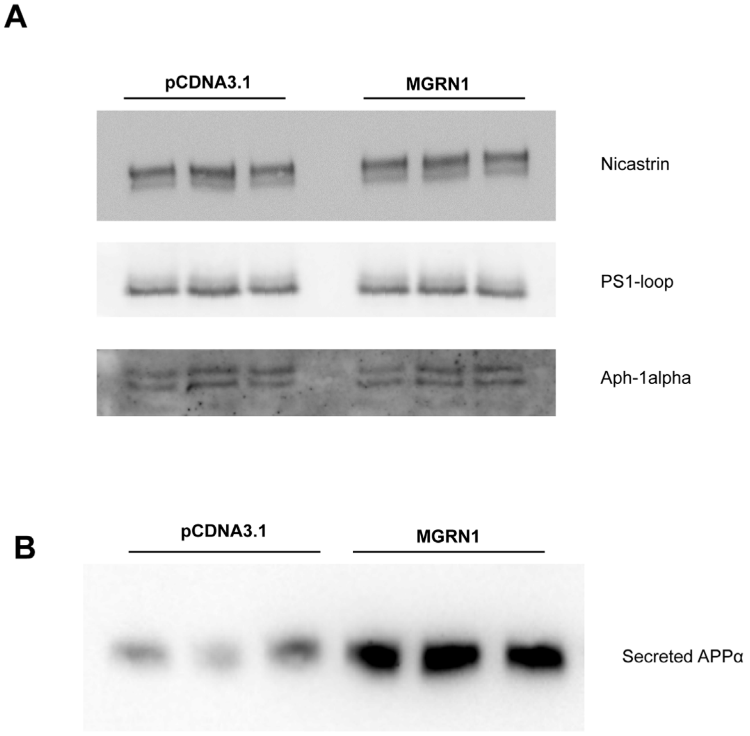

Supplementary Figure 1: Additional characterization of APP processing. (A) HEK293 cells stably overexpressing human wild type APP695 were transfected either with empty plasmid (pCDNA3.1) or with MGRN1 plasmid, and then their membrane extracts were analyzed for the expression levels of the gamma-secretase complex members Nicastrin (upper panel), Presenilin-1 (central panel) and Aph-1alpha (lower panel). No evident difference in their expression levels was detected between mock- and MGRN1-transfected cells (N=3). (B) Conditioned media of mock- and MGRN1-transfected HEK293 cells stably overexpressing human wild type APP695 were analyzed for their content in soluble APPα with 6E10 antibody. All conditioned media were normalized to total protein levels. An increase in sAPPα was detected in the conditioned media of MGRN1-transfected cells compared to mock-transfected cells conditioned media (N=3).
